# Using Specialist Screening Practitioners (SSPs) to increase uptake of the Bowel Scope (Flexible Sigmoidoscopy) Screening Programme: a study protocol for a feasibility single-stage phase II trial

**DOI:** 10.1186/s40814-016-0093-8

**Published:** 2016-09-14

**Authors:** Lesley M. McGregor, Hanna Skrobanski, Hayley Miller, Mary Ritchie, Lindy Berkman, Stephen Morris, Colin Rees, Christian von Wagner

**Affiliations:** 1 Cancer Research UK Health Behaviour Research Centre, Department of Epidemiology and Public Health, University College London, London, WC1E 6BT UK; 2 Gateshead Health NHS Foundation Trust, Queen Elizabeth Hospital, Queen Elizabeth Avenue, Sheriff Hill, Gateshead, Tyne and Wear NE9 6SX UK; 3 Patient representative, London, UK; 4 Department of Applied Health Research, University College London, London, WC1E 6BT UK; 5 South Tyneside NHS Foundation Trust, South Tyneside District Hospital, Harton Lane, South Shields, Tyne and Wear NE34 0PL UK

**Keywords:** Patient navigation, Bowel scope screening, Colorectal cancer, Non-attenders

## Abstract

**Background:**

The NHS Bowel Scope Screening (BSS) programme offers men and women aged 55 years a once-only flexible sigmoidoscopy (FS), a test that can help reduce colorectal cancer (CRC) incidence and mortality. However, the benefits of BSS are contingent on uptake. This National Institute for Health Research-funded single-stage phase II trial will test the feasibility of using patient navigation (PN), an intervention that offers support to patients to overcome barriers to healthcare, to increase BSS uptake within a socially deprived area of England.

**Methods/design:**

All individuals invited for BSS at South Tyneside NHS Foundation Trust during the 6-month recruitment period will be invited to take part in the study. Consenting participants will be randomised to receive PN or usual care in a 2:1 ratio. PN involves non-attenders receiving a phone call from a Specialist Screening Practitioner (SSP) who will elicit reasons for non-attendance and offer educational, practical, and emotional support as needed. If requested by the patient, another appointment for BSS will then be arranged. We anticipate 30 % of participants will be non-attenders. Using A’Hern single-stage design, with 20 % significance level and 80 % power, at least 35 participants who receive PN need to subsequently attend for PN to be considered worthy of further investigation in a definitive trial. The primary outcome measure will be the number of participants in the PN group who re-book and attend their BSS appointment. A qualitative analysis of the PN transcripts, and interviews with the SSPs, will also be conducted, alongside a quantitative analysis of completed patient-reported experience questionnaires. An economic analysis will calculate the costs of delivering PN.

**Discussion:**

This feasibility study will be instrumental in deciding whether to conduct the first definitive trial of PN in BSS in England. If PN is subsequently shown to be cost-effective at increasing uptake of BSS, NHS policies could be modified to implement PN as a standard service. The results will be disseminated in peer-reviewed journals and at scientific conferences.

**Trial registration:**

International Standard Randomised Controlled Trial Number, ISRCTN13314752

**Electronic supplementary material:**

The online version of this article (doi:10.1186/s40814-016-0093-8) contains supplementary material, which is available to authorized users.

## Background

Colorectal cancer (CRC) is the fourth most common cancer and the second leading cause of cancer death in England [1]. CRC screening can help detect cancer early, when it is more easily treated, subsequently helping to reduce CRC mortality rates [2, 3]. In England, the National Health Service (NHS) runs an organised population-based CRC screening programme (Bowel Cancer Screening Programme, BCSP) which offers biennial screening via guaiac-based faecal occult blood testing (gFOBt) to men and women aged 60–74. In March 2013, the BCSP additionally started the national roll-out of the ‘Bowel Scope Screening (BSS) Programme’, which offers men and women a single flexible sigmoidoscopy (FS) examination at age 55. The UK FS trial has shown that a single FS examination, with its capacity to remove any pre-malignant growths, reduces CRC mortality by 43 % and incidence by 33 % [4].

Similar to all cancer screening programmes, the benefits of BSS are largely dependent on high uptake; in the current BCSP using gFOBt, only 54 % of people invited to do the gFOBt return a completed kit [5]. Findings from a meta-analysis and a pathfinder study suggest that uptake of BSS is likely to be as low as gFOBt, undermining the potential of the programme [6, 7]. Analyses of uptake data from the first 14 months of the BSS programme roll-out in six pilot centres indicated uptake to be only 43.1 % [8]. BSS participation has also been found to be strongly socially graded, ranging from 32.7 % in the most deprived to 53.2 % in the least deprived area quintiles, resulting in an urgent need to develop effective interventions to promote BSS uptake, particularly among lower SES groups.

Patient navigation (PN) involves specially trained individuals giving tailored support to patients to overcome barriers personally preventing them from optimising their healthcare along the cancer pathway. PN can take on many communication formats, e.g. face-to-face or over the telephone, and includes the delivery of clear information and practical guidance and advice to the individual, in an emotionally supportive context [9]. PN has been shown to increase cancer screening participation in the USA, including CRC screening [10–13]. It is particularly effective among ‘hard-to-reach’ groups but has also been effective in the general population. A recent trial found benefits associated with PN over-and-above providing an organised screening programme [13]. In the UK, PN has currently only been employed in a breast cancer screening context, with results showing an increase in uptake among African Caribbean women, in two socially diverse areas of London [14].

Incorporating PN within a CRC screening context in the UK, and providing a more personal and interactive approach to communicating information about BSS, may circumvent many barriers to CRC screening. Evidence from the gFOBt screening programme suggests that older adults, particularly those with low literacy, have difficulty in extracting what they need from the current information materials [15–17]. There is also a lack of public awareness of CRC as a common cancer and of the primary aim of BSS in preventing rather than diagnosing CRC [18].

Logistical barriers to preparing for, and participating in, hospital-based BSS appointments have also been identified. For example, difficulties have been found in comprehending instructions for home-administered enemas for bowel preparation, particularly for those with low literacy, even though it is reasonably acceptable among the public [19, 20]. There are also some well-recognised emotional barriers including fear of the test and cancer diagnosis and fatalistic attitudes [21]. Helping to manage feelings and fears associated with cancer is one of the main tasks addressed by patient navigators alongside access to care and other logistical issues (e.g. transportation) [9, 22].

This pilot trial will test the feasibility of using a PN intervention to increase uptake of BSS in a socially deprived area in the North East of England. The intervention will involve Specialist Screening Practitioners (SSPs) giving tailored support to individuals who do not attend their appointment for BSS.

SSPs are well situated to deliver PN as they are already highly knowledgeable about BSS and the BCSP more generally, regularly communicating with patients the risks and benefits of screening and supporting patients through their screening procedures (FS or follow-up colonoscopy). The high attendance rates among those requiring follow-up investigations after gFOBt [23], and the high levels of patient satisfaction, highlight the effective educational and pastoral achievements of SSPs in the screening programme (Vart G, Marshall S, Nickerson C, Rees C, Wardle J, Von Wagner C: Patient reported experiences among patients requiring diagnostic follow-up in the English Bowel Cancer Screening Programme, unpublished).

The primary objective of this study is to assess the feasibility of incorporating PN for patients who do not confirm or attend their BSS appointment. The results will inform the design of a future large-scale randomised controlled trial (RCT), to compare whether a PN intervention is more effective at increasing uptake of BSS than the ‘usual care’. The feasibility of the PN intervention will be determined by measuring the number of people who provide their contact details (recruitment rates), the acceptability of PN from the perspective of patients (patient-reported experience questionnaires) and staff (exit interviews with SSPs and the Research Nurse), and BSS uptake rates of those navigated. The costs of delivering PN will also be calculated, and we will plan the economic evaluation that would accompany the subsequent RCT.

## Methods/design

### Design

**Fig. 1:**
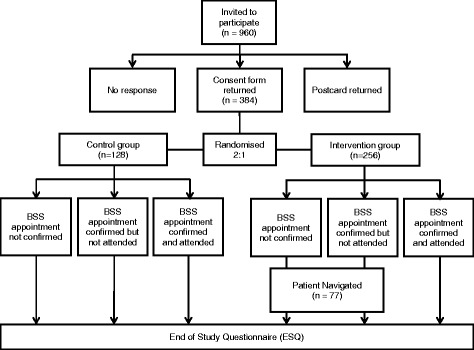
CONSORT flow diagram outlining the study procedure (with estimated numbers)

A single-stage phase II trial will test whether PN is likely to meet the basic level of efficacy (in this case by increasing uptake), is practical and acceptable within a UK setting and is therefore worthy of further investigation in a large-scale RCT [24]. To enhance the opportunity to conduct PN with individuals who do not confirm or attend their appointment, while keeping SSP workload to a minimum, a randomisation ratio of 2:1 will be used in favour of the intervention group (vs. usual care). The aim of the trial is not to make a direct comparison between the intervention and control groups but rather to consider the level of uptake in the PN group, while also assessing patients’ acceptability and willingness to be randomised. The CONSORT diagram is shown in Fig. 1.

The design of this protocol has followed the recommendations of the Standard Protocol Items: Recommendations for Interventional Trials (SPIRIT) guidelines [25, 26]. A full copy of the SPIRIT guidelines checklist can be found in Additional file 1.

### Recruitment and setting

**Fig. 2:**
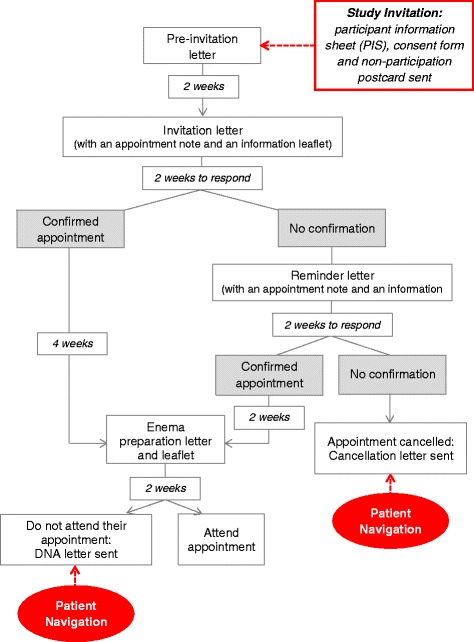
Positioning of the intervention within the structure of the BSS programme

Recruitment will be conducted through the South of Tyne Screening Centre. The Screening Centre operates across three sites with one site, South Tyneside District Hospital (STDH), involved in this current study. Approximately, 40 people each week are invited to have BSS at STDH. All individuals invited for BSS at STDH will also be invited to take part in this study (see Fig. 2). There are no study exclusion criteria; however, only individuals who return a study consent form with their contact details, including telephone number(s), will be able to participate in this study. Recruitment will occur over a 6-month period.

### The intervention

The PN intervention will consist of at least one telephone call that will begin with the SSP introducing themselves and the purpose of the call. The SSP will use open-ended and non-threatening questions to elicit reasons for the patient’s non-attendance/non-confirmation and their motivation to attend BSS in the future. If the patient appears interested in BSS, the SSP will reiterate its benefits and offer solutions to any identified barriers (e.g. an earlier appointment time or assistance with the enema). In the role of navigator, the SSP should provide two types of support: practical/instrumental and emotional/relationship [27]. The SSPs will receive training on what PN is and how it should be delivered during this study. A manual will be developed to supplement the training and provide a resource for SSPs. The training will be assisted by a patient representative who will conduct role play scenarios and provide feedback to the SSPs. The navigator should refer to the manual for instrumental support options but use their communication and interpersonal skills to offer emotional support and strengthen the relationship between the hospital and the patient.

### Procedure

The Screening Centre hub, where all BSS invitations are packed and prepared for mail out, will oversee the study invitation process. During the recruitment period, the hub staff will insert a study invitation envelope to the standard BSS pre-invitation letter sent to all eligible individuals. The BSS pre-invitation letter briefly introduces BSS and forewarns of an additional letter being sent in a further 2 weeks, which will formally invite the individual to attend a BSS appointment at a specific date and time (see Fig. 2 for the BSS invitation process). The study invitation envelope will contain a participant information sheet (PIS), a consent form, a pre-paid return envelope, and a non-participation postcard. The PIS will provide information about the aim of the research study and detailed information about the consent process, data protection and study procedure, including the telephone number that will show on their caller display if they were to receive a call from an SSP. It will be made clear that the decision to participate in the study is separate to the decision to take part in screening. Each consent form will have a unique study ID attached to it, and hub staff will note which study ID corresponds to each person invited. To take part in the study, participants will be asked to complete and return the consent form to the Research Nurse (RN) at STDH in the return envelope provided. Those not wanting to take part in the study will be invited to indicate their reason on the non-participation postcard and return it freepost to the researchers.

By offering consent, participants will be agreeing to share their contact details with the assigned RN and to be potentially contacted by a SSP. The RN will contact the hub staff to confirm the NHS number of those consenting to take part in the study, using the unique study ID. Once the RN has the NHS numbers, they can then use the web-based national Bowel Cancer Screening System (BCSS) within STDH to follow each participant along the BSS pathway and obtain their BSS confirmation and attendance status.

On receipt of each consent form, the RN will consult a pre-generated randomisation list to determine which study group each participant should be allocated to. Two randomisation lists (one for each gender) will be generated by a medical statistician, who will randomly allocate participants to receive either PN or usual care with a ratio of 2:1 favouring the intervention. It is not possible to blind the RN and SSPs to each participant’s study group allocation. However, the participants themselves will only know the group they have been allocated to when they receive either a navigation phone call or their end-of-study questionnaire (ESQ): the ESQ will be accompanied by a cover letter thanking them for their participation and informing them of their group allocation.

The RN will work with University College London (UCL) researchers and SSPs and use the BCSS to document all activities of consenting participants on a database devised for this study (e.g. group assignment, original appointment date and subsequent changes made, attendance status, date of ESQ mail out, and date of receipt of completed ESQ). This will allow the RN to monitor who is eligible for PN and to obtain important information for establishing the feasibility of the study design and expectations for the larger RCT.

#### Usual care group

As per the standard BSS process (see Fig. 2), individuals in the usual care group will receive an invitation to take part in BSS 2 weeks after receiving the pre-invitation letter (and study information envelope). The BSS invitation letter includes details of an assigned appointment date and time (8 weeks in advance). The letter also requests confirmation of the appointment. If no confirmation is received from the patient within 2 weeks of the invitation going out, a reminder invitation letter is sent. If confirmation is still not received within 2 weeks of the reminder, a ‘cancellation’ letter is sent and there is no further contact from the Screening Centre. If confirmation is received, an enema with preparation instructions is sent approximately 2 weeks before the appointment date. Patients who confirm but then fail to attend their appointment receive a ‘did not attend (DNA)’ letter.

#### Intervention group

Individuals in the intervention group will also follow the above usual care process. However, if an individual either fails to confirm or attend a confirmed appointment (‘non-attenders’), they will, in addition to the cancellation/DNA letter, enter a navigation episode, whereby a SSP trained in PN will make up to three attempts to contact the individual by telephone. All call attempts will be documented and, if contact is made, the SSP will establish the reasons for non-attendance, offer support and, if suitable, arrange a new appointment. The navigation episode could involve multiple telephone conversations and closes when a decision to arrange a new BSS appointment, or not, is made. Participants in the PN group will only be eligible for one navigation episode, i.e. if a further BSS appointment is made following PN, but is not attended, only a further ‘DNA’ letter is sent, as per standard practice: no additional navigation call attempts will be made.

**Fig. 3:**
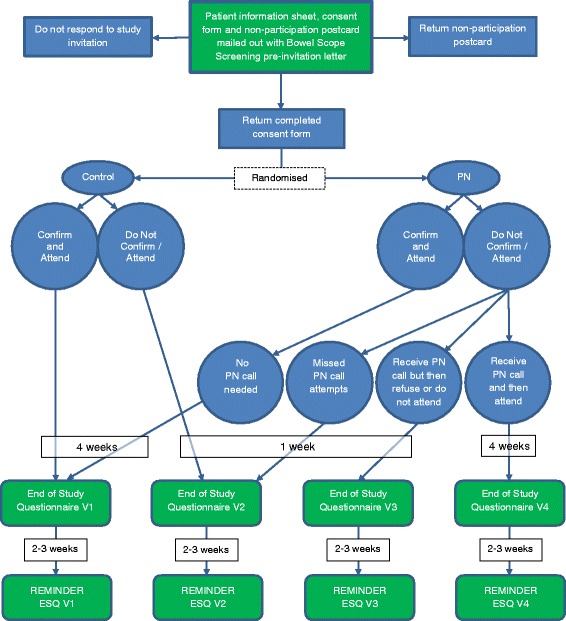
Flowchart of study materials

All consenting participants will be sent an ESQ, with a freepost-return envelope, either 4 weeks following an attended appointment or 1 week after a non-attended appointment or final PN call (see Fig. 3). There will be four versions of the ESQ with questions varying depending on the participant’s study group and BSS attendance status. The aim of the ESQ is to assess participants’ knowledge of BSS and their satisfaction with their choice to attend screening or not. The ESQ for those receiving PN also aims to find out how participants felt about this process (e.g. their perceptions of the usefulness of PN and how it made them feel) and the effect PN has on various psychosocial variables such as informed choice and BSS knowledge [28–36]. If ESQs are not returned within 2 weeks, a reminder ESQ with a freepost-return envelope will be sent.

Additionally, at the end of the study, qualitative interviews will be conducted with the SSPs, to allow an evaluation of the barriers and facilitators for implementing PN at the organisational level, exploring how PN affected SSP’s workload and processes in the wider department.

### Sample size

Currently, there is no directly comparable evidence to estimate the number of people who will consent to take part in this study. Because our sample is likely to consist of people who support the BSS in principle, we have used recruitment figures from the UK FS trial as a guide to estimating our recruitment rate.

South of Tyne Screening Centre will invite approximately 960 people for BSS at STDH during the recruitment period. In the UK FS trial, 55 % of those initially approached were interested in receiving a FS examination [37]. Given that people in this study will receive an invitation for BSS regardless of their response to our invitation, recruitment may be lower (i.e. 40 %; *N* = 384).

It is likely that uptake among this self-selected sample would be higher than for the general population. On the basis of the UK FS trial, we predict that about 70 % of the sample would attend their original appointment as part of the usual pathway. The remaining 30 % of consenting participants would fail to attend their appointment (*N* = 116) of whom we would allocate two thirds (*N* = 77) to receive PN.

Using A’Hern single-stage design, with 20 % significance level and 80 % power, at least 35 participants would need to re-schedule and subsequently attend their appointment for PN to be considered worthy of further investigation [24].

### Outcome measures

The primary outcome measure of this study will be the number of non-attenders in the PN group who are navigated and then re-book and attend their new BSS appointment.

Additional outcomes measures will include the following:

- Barriers to BSS attendance: All PN sessions will be audio recorded (with patient permission) and transcribed. An analysis of the transcripts will aim to highlight all the barriers to attendance described by patients and the extent to which they are modifiable.
- An evaluation of the PN process: Approximately seven SSPs will be assigned to this study. SSPs will monitor the number of contact attempts made and the duration of telephone calls made within each navigation episode. The audio recordings of the navigation calls will also be analysed with regard to the SSPs adherence to the PN manual, the type of support given (e.g. practical and/or emotional), and the action plans agreed with patients. Exit interviews with SSPs will be conducted, audio recorded and analysed with the aim of ascertaining their views on the PN process. Exit interviews will either be conducted face-to-face within the hospital setting or over the telephone, whichever is most convenient for each SSP.
- Patient-reported outcomes: Completed ESQs will be analysed to ascertain the number of people making an informed choice in both groups and, where relevant, satisfaction with the BSS procedure and PN intervention.
- Reasons for not participating in the study: A brief freepost-return A5 postcard will be provided within the study invitation envelope that will be sent alongside the BSS pre-invitation letter. The postcard will give those not wanting to take part in the study the option to choose among reasons for non-response/refusal (e.g. ‘I do not want to share my personal details with the researchers’, ‘I did not think I will need any support’), with responses considered in the development of the RCT.
- Economic analysis: We will calculate the cost of delivering the PN intervention, including SSP time, and training. We will also plan the economic evaluation that would accompany the full RCT.

### Planned analyses

#### Primary analysis

The overall recruitment and uptake rates by trial arm will be described; the study is not powered to make a direct comparison between arms but will help inform sample size requirements for a phase III trial.

#### Qualitative analysis

Transcripts of the PN telephone calls and interviews with SSPs will be analysed separately and managed using QSR International’s NVivo 10 Software. Framework analysis will be applied to the PN telephone call transcripts, with aspects such as the barriers highlighted, advice given, and outcomes achieved extracted for review [38]. SSP interviews will be analysed using thematic analysis with an inductive approach as we aim to gain insight into the views of SSPs with regard to the intervention’s concept and design, and their experience of intervention delivery [39]. One member of the research team will lead the analysis with a further two research team members involved in discussions to verify the results. A sub-sample of anonymised transcripts will be read by our patient representative to check interpretations by the main research team. The telephone transcripts will also be matched to the corresponding SSP interview transcript to give context to the interviews and triangulate the data.

#### Quantitative analysis

Data collected on the study database and from returned non-participation postcards will be analysed and mainly reported as descriptive data (e.g. attendance rates, time for return of ESQs, number of people selecting specific reasons for non-study participation). The ESQ data will be analysed using a mixture of descriptive and inferential statistics including chi-square analysis and linear and logistic regression so that predictors of attendance and non-attendance can be investigated, as can between group differences in views, attitudes, knowledge and satisfaction with BSS. Results will be presented with a 95 % confidence interval.

#### Economic analysis

A detailed analysis of the cost of PN versus usual care will be undertaken. First, the analysis will include the costs of SSP training and time, plus other costs during the navigation pathway. Second, we will calculate the impact of PN on subsequent bowel cancer screening and diagnosis. Health service contacts will be collected by the UCL researchers during the study from patients, plus the routinely recorded data within the BCSP, and priced using national unit cost data. Third, we will undertake a feasibility study for a full economic evaluation of PN versus usual care. The aim is to plan the economic evaluation that would accompany the definitive RCT. We will develop a plan to estimate the lifetime incremental cost per quality-adjusted life year (QALY) gained of the intervention in the definitive RCT. In the present study, we will identify the following: (a) the main NHS/Personal Social Services (PSS) cost components; (b) the resource use and unit cost data required for each of these cost components and how best to source these data; (c) potential sources of health-related quality of life data suitable for estimating QALYs in this patient group and, if primary data collection is required, how best to do this using pre-existing instruments such as the EuroQol-5 Dimensions Scale (5 level; EQ-5D-5L) (Kind P: The EuroQol instrument: an index of health-related quality of life, unpublished); (d) the potential sources that could be used to estimate residual life expectancy and other long-term outcomes among patients; and (e) the non-NHS/PSS impacts that are important to record (e.g. days off work) and how best to obtain these.

### Trial status

Data collection is ongoing but the randomisation ratio has now been increased to 4:1 in favour of the intervention, following lower than expected consenting rates within the first 3 months of recruitment.

## Discussion

BSS offers the unique benefit of reducing CRC incidence as well as mortality, but this benefit can only be realised through uptake of the procedure. Within the first 14 months of the BSS programme’s launch, 21,187 invitations were sent out to eligible people across six pilot centres in England and the overall uptake was 43 % [8]. Although this was higher than expected in the early stages of the programme’s national roll-out, a need to proceed with interventions to boost uptake and reduce socioeconomic inequalities was highlighted [8]. Within the South of Tyne Screening Centre specifically, a below average uptake of only 37 % was reported, promoting itself as a key centre in which to base intervention initiatives.

Incorporating PN within the NHS BCSP, and providing a more personal and interactive way of communicating information about BSS, seems likely to circumvent many of the barriers associated with CRC screening. As part of their role as patient navigator, SSPs will have the opportunity to communicate the benefits of BSS more effectively and help to manage the feelings and fears associated with cancer screening and diagnosis, alongside access to care and other logistical issues. While navigation has been shown to work in the USA, its integration into the UK system requires investigation not only from a theoretical standpoint but also a practical one. This study not only aims to assess the acceptability of PN but also how it can be implemented within the system.

### Potential limitations, challenges and risks

Unfortunately, the telephone numbers of people eligible for BSS are not readily available within the programme and are only provided to SSPs through the appointment confirmation process. For this reason, and to adhere to research ethical standards, we will have to obtain consent to take part in this study and, as part of the consent process, specifically request the provision of an individual’s telephone number(s). It is possible that the consent process will represent a barrier and ultimately restrict the intervention to more ‘activated’ individuals, thereby introducing a selection bias. However, we have taken this into consideration and have assumed a higher uptake rate (70 %) within our sample compared to the general population.

Furthermore, there is a risk that people will mistake the consent for the study as confirmation of their future attendance at a BSS appointment which could negatively affect the BSS invitation process. As a result, we will closely monitor the impact of the study invitations on BSS uptake.

An additional limitation is that the PN training session and related manual will be developed and delivered by non-PN experts, which may lower the extent to which SSPs accept and identify with the PN intervention and their confidence to perform it.

### Conclusion

The results of this feasibility study will be instrumental in deciding whether a proposal will be made to conduct a large-scale RCT of the PN intervention in the BSS programme, in England. If PN was subsequently shown to be cost-effective at increasing uptake of BSS, NHS policies could be modified to implement PN as a standard service. Another important part of this feasibility study will be to identify barriers associated with BSS. Disseminating the findings from this study will therefore facilitate the development of additional strategies to increase uptake of BSS.

## Additional file

Additional file 1: SPIRIT 2013 Checklist: Recommended items to address in a clinical trial protocol and related documents. (DOC 120 KB)

## Abbreviations

BCSP: Bowel Cancer Screening Programme
BSCC: Bowel Cancer Screening System
BSS: Bowel Scope Screening
CRC: Colorectal cancer
DNA: Did not attend
EQ-5D-5L: EuroQol-5 Dimensions Scale (5 level)
ESQ: End-of-study questionnaire
FS: Flexible sigmoidoscopy
gFOBt: Guaiac faecal occult blood test
ISRCTN: International Randomised Controlled Trial Number
NHS: National Health Service
PIS: Participant information sheet
PN: Patient navigation
PSS: Personal Social Services
QALY: Quality adjusted life year
RCT: Randomised controlled trial
RN: Research Nurse
SSP: Specialist Screening Practitioner
STDH: South Tyneside District Hospital
UCL: University College London
